# Song variation of the South Eastern Indian Ocean pygmy blue whale population in the Perth Canyon, Western Australia

**DOI:** 10.1371/journal.pone.0208619

**Published:** 2019-01-22

**Authors:** Capri D. Jolliffe, Robert D. McCauley, Alexander N. Gavrilov, K. Curt S. Jenner, Micheline-Nicole M. Jenner, Alec J. Duncan

**Affiliations:** 1 Centre Marine Science and Technology, Curtin University, Perth, Western Australia, Australia; 2 Centre for Whale Research (WA) Inc., Fremantle, Western Australia, Australia; Oregon State University, UNITED STATES

## Abstract

Sea noise collected over 2003 to 2017 from the Perth Canyon, Western Australia was analysed for variation in the South Eastern Indian Ocean pygmy blue whale song structure. The primary song-types were: *P3*, a three unit phrase (I, II and III) repeated with an inter-song interval (ISI) of 170–194 s; *P2*, a phrase consisting of only units II & III repeated every 84–96 s; and *P1* with a phrase consisting of only unit II repeated every 45–49 s. The different ISI values were approximate multiples of each other within a season. When comparing data from each season, across seasons, the ISI value for each song increased significantly through time (all fits had *p* << 0.001), at 0.30 s/Year (95%CI 0.217–0.383), 0.8 s/Year (95%CI 0.655–1.025) and 1.73 s/Year (95%CI 1.264–2.196) for the *P1*, *P2* and *P3* songs respectively. The proportions of each song-type averaged at 21.5, 24.2 and 56% for *P1*, *P2* and *P3* occurrence respectively and these ratios could vary by up to ± 8% (95% CI) amongst years. On some occasions animals changed the *P3* ISI to be significantly shorter (120–160 s) or longer (220–280 s). Hybrid song patterns occurred where animals combined multiple phrase types into a repeated song. In recent years whales introduced further complexity by splitting song units. This variability of song-type and proportions implies abundance measure for this whale sub population based on song detection needs to factor in trends in song variability to make data comparable between seasons. Further, such variability in song production by a sub population of pygmy blue whales raises questions as to the stability of the song types that are used to delineate populations. The high level of song variability may be driven by an increasing number of background whale callers creating ‘noise’ and so forcing animals to alter song in order to ‘stand out’ amongst the crowd.

## Introduction

Baleen whales commonly use low frequency, high intensity sounds to communicate over large distances [1–3]. The purpose of these vocalisations remains uncertain and likely has multiple functions. In humpback whales the complex song structures are produced by males as part of reproductive displays [4–7], and it is likely that song serves some reproductive function in other baleen whale species as well [6–8]. Vocalisations are population specific with subpopulations defined by geographic range and song structure [9–13]. As such, the correct classification of song-types is important for successful acoustic monitoring of populations. The detection of specific song-types across protracted periods can indicate the migratory timing of individual whales or the population, and may allow an understanding of population structure and abundance [14]. Studying the communication of populations can provide clues as to the evolution of vocal systems and mechanisms for vocal learning within a population [6, 11, 15–19]. Changes to the vocal structure employed by a population can be indicative of large and small scale processes that shape vocal repertoires at the species and population level [17]. Changes in whale song may be in the form of the loss or addition of vocal elements or the modification of existing vocal elements [17, 20, 21]. Changes to existing vocalisation structure can be defined as changes to the duration and timing of song intervals, or composition and frequency changes in elements of the song [17] above what normal variation can be expected. A number of factors are thought to be responsible for shaping changes in song structure including physical processes such as increases in ambient noise, social changes such as cultural drift within the population, or through genetic drift [1, 13, 22, 23]. It is often unclear whether small scale variations in song structure are part of population wide changes or can be attributed to individual whales [24, 25]. A better understanding of the driving factors behind changes to vocal repertoires may provide clues as to the purpose of particular vocal signals, such as whether they have a reproductive or social context [7, 26–29]. It is thought that vocalisations within a familial group with a social context are least susceptible to change whilst those songs with a reproductive context are most likely to change [6, 27, 28, 30].

Population specific vocalisations are useful in the monitoring and management of cryptic or offshore species such as the Australian pygmy blue whale population (*Balaenoptera musculus brevicauda*) or termed here, the South Eastern Indian Ocean (SEIO) pygmy blue whale, a part of which traverses the Western Australia coast each year [31–33]. The use of passive acoustic monitoring (PAM) for assessing the abundance of pygmy blue whales in a quantitative fashion requires knowledge of the vocal repertoire, song structure and natural variability in the cue rate or proportion of animals vocalising. Blue whale song-types are categorised based on differences in song phrasing, inter song interval (ISI) or the time between phrase repetitions, unit frequency, duration, modulation or total song length [12, 24, 34, 35]. This paper explores the considerable on-going song variability found in the SEIO pygmy blue whale song (referred to as pygmy blue whale hereafter for brevity). This variability in song structure has implications for passive acoustic census techniques and for understanding the social and external features which may drive song function, structure and variability. Relative abundance estimates are derived from passive acoustics data using some measure of song production per unit time across seasons. Underlying these measures is the assumption that song production, structure and song repeat intervals are persistent over years, but this is not quite the case, as demonstrated by this study.

## Methods

**Fig 1:**
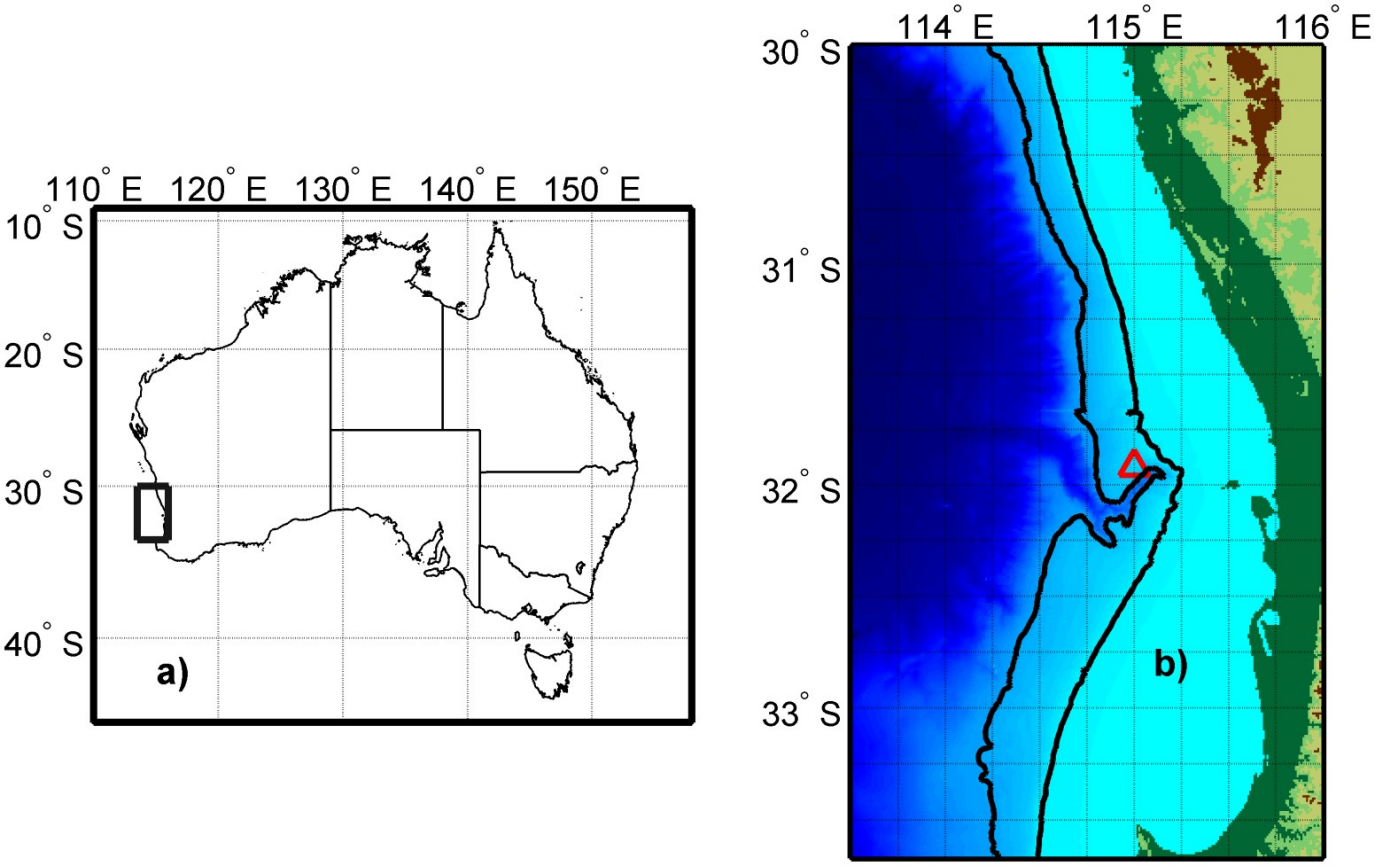
Location of Perth Canyon sampling area. The area sampled within Australia is shown by the rectangle in a), the Perth Canyon is shown on b), centred at 32° S 115° E, the general area of sea noise moorings is shown by the red triangle and the 1000 and 200 m depth contours are shown by the black curves (west and east curves respectively). Bathymetry from [38].

Long term data was collected from a passive acoustic observatory located in the Perth Canyon area to the north-west of Rottnest Island by Curtin University or as part of the Australian, Integrated Marine Observing System (Fig 1). Data was collected under Curtin University Animal Ethics Committee permit AEC_2013_28—Passive acoustic recording of marine animal (mammal and fish) vocalisations. Permits for deploying sea noise recorders were not required. Each passive acoustic observatory consisted of one to four Curtin University CMST-DSTO sea noise recorders (see www.cmst.curtin.edu.au\products or [36] for instrument and deployment details) set over 2003 to 2017 (Table 1). On occasion three or four instruments were deployed simultaneously in a tracking configuration, with three instruments in an approximate equilateral triangle of 5 km sides, and a fourth recorder in the triangle centre. The noise recorders were deployed on the seabed at a depth of 430 to 490 m. The recorders were set to collect sea noise samples of between 200–500 s every 900 s at a sample rate of 6 kHz with a low pass anti-aliasing filter at 2.8 kHz and a roll off applied below 8 Hz to flatten the sea noise spectra and so increase the effective dynamic range. All instruments were calibrated using white noise injection with the hydrophone in series to the noise generator output, allowing the full system frequency response to be corrected for in post processing (2 Hz to anti-aliasing filter frequency). The system clocks were set to UTC time before deployment and the clock drift was measured after recovery, allowing absolute time accuracy of ± 0.25 s, this driven by the jump in water temperature on deployment and recovery (see [36] for calibration details). Sea noise recorders were deployed for between eight and twelve months at which point they were retrieved in order to upload data and change batteries. When using hydrophone arrays for passive acoustic tracking, the accuracy of sound source localisations depends largely on the accuracy of the hydrophone positions and internal clocks. The GPS locations of the touch-down positions were recorded upon deployment of the hydrophones while instrument clocks were synchronised in accordance with the procedures outlined in [37].

**Table 1:** Details of sea noise logger primary deployments.

Set	Lat. (° ' S)	Lon. (° ' E)	Start	End	Len (s)
2615	31° 53.77’	115° 1.00’	18-Feb-2003	07-Jun-2003	205.3
2656	31° 50.86’	114° 59.92’	26-Feb-2004	14-Jun-2004	205.3
2672	31° 52.12’	115° 0.04’	30-Dec-2004	08-Jul-2005	205.0
2724	31° 54.08’	115° 1.14’	01-Jan-2007	25-Apr-2007	204.9
2802	31° 53.86’	114° 59.73’	26-Feb-2008	21-Apr-2008	204.9
2823	31° 54.47’	114° 59.08’	24-Feb-2009	11-Oct-2009	512.1
2884	31° 55.04’	115° 1.86’	13-Nov-2009	22-Jul-2010	460.9
2962	31° 54.14’	115° 1.61’	06-Aug-2010	08-May-2011	409.7
3006	31° 51.98’	115° 0.05’	14-Jul-2011	18-Jun-2012	307.3
3007	31° 53.07’	114° 59.96’	14-Jul-2011	16-Jun-2012	307.3
3004	31° 54.35’	115° 1.54’	14-Jul-2011	19-Jun-2012	307.3
3154	31° 53.05’	115° 0.81’	10-Aug-2012	14-Jun-2013	306.3
3376	31° 50.53’	115° 0.82’	28-Nov-2013	03-Nov-2014	307.3
3445	31° 52.66’	115° 0.66’	05-Jan-2016	30-Dec-2016	307.3
3444	31° 51.77’	115° 1.74’	23-Sep-2016	26-Aug-2017	307.3

Listed are: set number; Latitude (degrees and minutes S); longitude (degrees and minutes E); start day (UTC); end day (UTC); and sample length (s). All sets used a 6 kHz sample rate.

All samples were repeated every 15 minutes. Only one of the instruments used in the tracking grids is included.

Data analysis for this paper focused on the northern migration of pygmy blue whales from February to June, coinciding with peaks in acoustic presence in the Perth Canyon. Logger deployments from all sample years cover these peak months making data suitable for comparison between years (Table 1).

All data sets were initially checked for major noise sources using an approach where 5–18 day spectrograms were produced and dominant sources identified [39]. Detection algorithms for pygmy blue whale signals were run across all data sets and the outputs of these manually checked. During the checking process the presence of all source types was noted.

**Fig 2:**
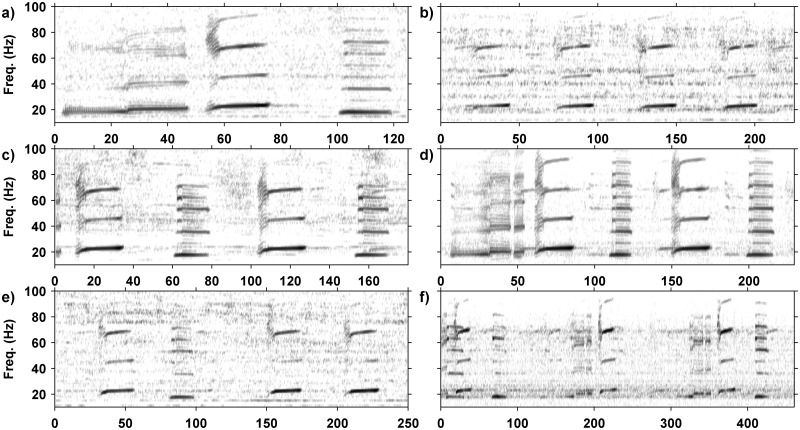
Spectrograms of pygmy blue whale song variants. All spectrograms made with 1024 point Fast Fourier Transform, 0.8 overlap using a 1 kHz sample rate (0.977 Hz and 0.205 s frequency and time resolution, respectively). The x-axis is time, in s, the y-axis is common for all panels. Shown are: a) one version of the normal, three unit song (P3) with the Type I (0–50 s), II (50–75 s) and III (100–125 s) units; b) the ‘song’ which repeats only the type II unit (P1); c) the song which repeats only the type II and III units (P2); d) a *P3A* song-type displaying a three unit song sequence followed by a two unit song sequence; e) a *P2B* song structure showing a two unit sequence followed by a lone type II unit and then another type II unit which marks the start of the next sequence; and f) a *P3B* song-type showing a three unit song sequence followed by a type I and type II unit and then another complete three unit phrase.

Pygmy blue whale songs were detected using a search algorithm initially defined in [5], which searches for the fundamental frequency of 20–23 Hz and the third harmonic of 60–70 Hz of the type II unit in the three unit pygmy blue whale phrase-type as shown in Fig 2 [12, 33, 35, 40]. The type II unit of the pygmy blue whale song was present in all song varieties. The detection algorithm had miss-detection and false-detection rates of less than 5% as described in [5]. The search algorithm isolated the signal by locating the frequency sweep based on a multivariate analysis of spectrogram features of the recorded signal [5]. In many data sets (all from which manual song analysis was carried out) each detection was checked manually by viewing the spectrogram with detections marked. Song structure, duration and frequency were analysed for each detection where signals could be easily isolated from surrounding noise. Where continuity of song structure, duration, the animal’s track (where available) and the ISI of songs was observed within a single recording, the signals were assumed to be produced by the same vocalising animal.

**Fig 3:**
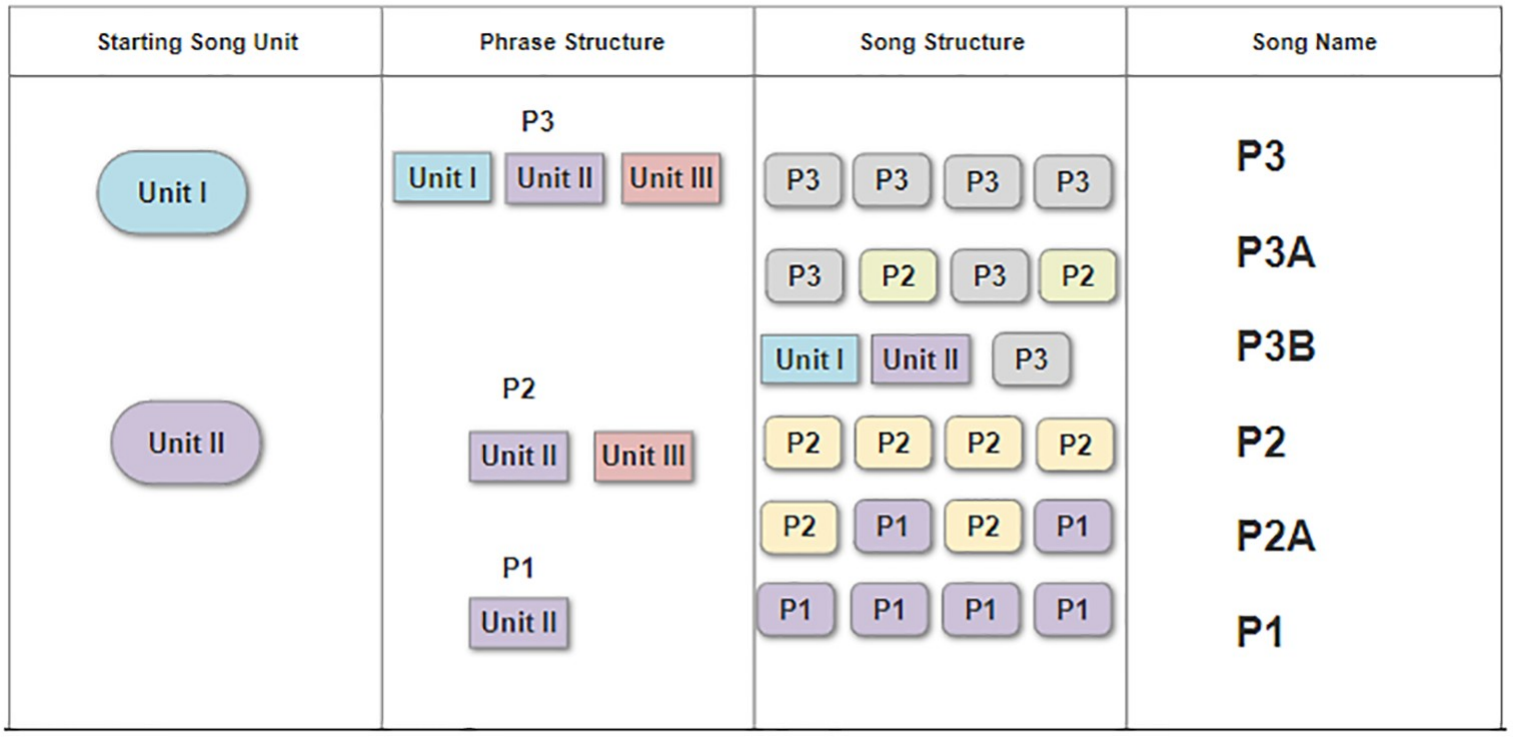
Song structure classification. The SEIO pygmy blue whale song type has three defined units combined in different sequences to produce phrases that are then linked together in song. Phrases are categorised by the order that units appear with temporal variations in the length of phrase types. Songs are generally composed of the same phrase repeated over and over, though combination songs comprised of two different phrases in sequence have been observed.

Classification of phrase structure was carried out using two approaches, the first was manually based by viewing spectrograms, and identifying the phrase based on hierarchical structure and presence or absence of particular units, as shown in Fig 3. The pygmy blue whale song type has three units, type I, II and III as shown on Fig 2a. The sequence in which these units were repeated was used to classify phrase structure, and where repeated phrases were available for analysis, song structure was classified. For most songs, only one phrase structure was repeated in sequence, though combinations of phrase structures were repeated in hybrid song types. A *K* means cluster analysis was used in the R statistical environment to sort song events based on the type and order of the first three song units. Clustering analysis was run with a set seed of 20, and six categories. The resulting analysis sorted 3,239 song events into the six song structures with 100% accuracy. A song catalogue was subsequently produced describing each of the three known song units, the five phrase variations including three structural variations and two temporal variations, and six song sequence variations. The validity of this catalogue for classification of SEIO pygmy blue whale song was assessed using an inter-rater reliability test, an established protocol for classification of cetacean vocalisations [41–44]. A randomly selected subset of 22 signal spectrograms displaying different phrase and song variations was presented to five untrained observers. Spectrograms were produced with a 1024 FFT, Hanning window with no overlap, and 1 kHz sample rate. Each spectrogram was presented on a single slide and viewed by the observers in PDF format. All observers were supplied with a hard copy of the SEIO PBW song catalogue and asked to classify the signals based on the appearance of the spectrographic contour, phrase organisation and duration [43]. A Fleiss unweighted Kappa analysis was used to statistically test the agreement of song classification between the five untrained observers [45].

The time between successive phrase production, or ISI, was measured from manually derived data to classify the phrase structure, although there was a bias here as periods of high numbers of simultaneously calling pygmy whales could not be analysed manually because of the difficulty of identifying individual singers. For this reason, manual analysis was only carried out on song events where a solitary animal was vocalising. For the manual processing, data was pre-processed in the MATLAB environment to calculate the power spectral density of sea noise for each sea noise sample using the software package CHORUS [39]. Spectrograms were produced using a 6000 point FFT and Hanning window with no overlap. Spectrograms of each sample were stacked and displayed in batches of 5–20 days for quick perusal for pygmy blue whale presence, then perused individually where pygmy blue signals were present.

**Fig 4:**
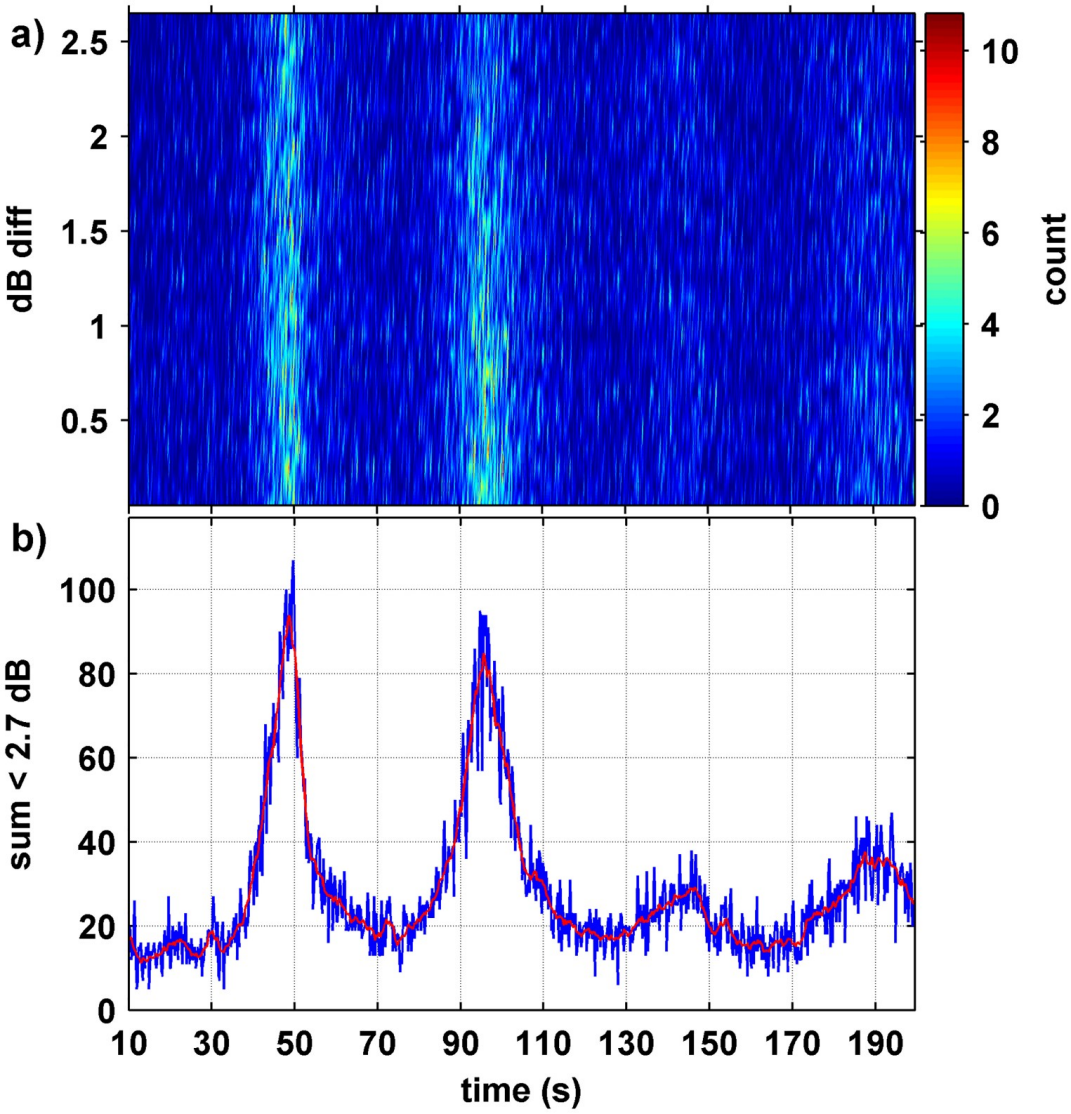
Inter-song-interval analysis. Density distribution (a) of song-to-song time and level differences for consecutive type II units, for 2016 with (b) the curve derived by summing data < 2.7 dB (blue is raw data, red is linearly smoothed data). Panel a) was derived using a 0.25 s and 0.1 dB time and level difference, respectively.

A second analysis approach was used to study the ISI values and relative proportions of song types across each season. This technique used all data available from the Perth Canyon (thirteen seasons over 2003–2017, with 2006 and 2015 not sampled, Table 1). A brief summary of this technique is listed below, details are given in S1 File. The search algorithm for locating the type II unit of pygmy blue whale songs was run across all Perth Canyon data sets, with the detector output of several data sets fully manually checked. Each pygmy blue whale detection (the type II unit) was assigned an arrival time within a sample using a consistent technique to define arrival time (the time at which 5% of the whale unit energy arrived) and the sound pressure level derived for the type II upper frequency unit, by band pass filtering the calibrated data. If more than one pygmy blue whale type II unit was present within a single sea noise sample, the difference of received level and arrival time of all combinations of type II units in that sample was derived. The same process was repeated for each sea noise sample and the time and level difference data assembled for all samples within each season. This gave a series of arrival time difference values (or potentially song repetition interval since the same unit in repeated songs may have been found) each with a level difference, for all type II to type II song unit combinations, for each season. The set of values was treated as a feature space and gridded for counts of unit-to-unit time and level differences which fell within set bounds, that is bounds of level and time differences were set and the number of values within these bounds counted (Fig 4a). One would expect that for the same animal vocalising within a sample, the level difference of the repeat type II unit (song ISI) would be within a few dB of the prior type II unit and that the same unit-to-unit time differences would be similar between phrases. Thus, common inter-song intervals, as given by the type II separation for individuals, would sum in the feature space at small level differences while the time and level differences for different animals would essentially be random noise and so not sum. This was the case and is observed on Fig 4.

By summing counts for values less than 2.7 dB of type II to type II unit level differences in the gridded feature space, curves of song repetition intervals were derived for each season in the Perth Canyon (termed ISI-curves, with an example shown in Fig 4b). Peaks in the ISI-curves occurred at the mean ISI for the respective phrase-type, and at multiple repetition intervals of the shorter phrase-types. The peak values in time windows of 30–60 s, 70–100 s and 160–200 s were found which gave the ISI for the three major phrase-types, noting that in Fig 4 the fourth peak at 140–150 s is a multiple of the first song type observed (ie the peak at 140–150 s represents the time between *P1* to *P1* to *P1* phrases, see S1 File for elaboration of how this is dealt with). The windows used to derive ISI values were relatively wide time spans as the ISI spacing was found to shift across years. The ISI-curve peaks were gentle and had some ‘noise’ in terms of small scale fluctuations associated with them. To remove this ’noise’ the ICI curves were smoothed, using a running, linear fit encompassing ± 5 points either side of the point in question, to give a smoothed value for that point. These smoothed curves were used for obtaining peak time and count values. A resolution of 0.1 s and 0.25 dB was used in the gridded feature space which derived these curves, with the respective x or y value for each ‘bin’ used to develop the 2D feature space, placed in the centre of the bin.

The magnitude of the ISI-curve peaks combined with the sea noise sample length also gave information on the proportion of song-types encountered each year. Details of how this was done are given in S1 File. Briefly the technique compared the magnitude of the peaks of the ISI-curves, after accounting for the expected number of calls of that ISI length in that sample length, the ‘noise’ and by accounting for multiples of a shorter ISI song type adding into a longer ISI song-type. Several models were built to verify how well the ISI analysis technique was able to predict data sets with different ratios of *P1*, *P2* and *P3* songs present, with root mean squared errors of input ratios compared with derived ratios at < 1%.

In order to better understand the movement patterns and presence of pygmy blue whales in the Perth Canyon area, vocalising animals were localised in space based on the time difference of arrival (TDOA) of the vocal signal at the noise recorders of the passive acoustic observatory. Tracking analysis could only be carried out for 2010, 2011 and 2012 where data sets had been collected successfully from four recorders within the observatory.

Localisation of pygmy blue whale vocalisations was based on the type II song unit as defined in [37]. Spectrogram correlation was used for TDOA estimates rather than waveform correlation in an attempt to lessen the effects of multipath propagation. The Levenberg-Marquardt least square method was used for localisation and is explained in detail in [37].

Whale localisation results were filtered to only include individual locations with errors of less than 0.5 km (an error ellipse was derived for each location). The filtered results were sorted into individual days and viewed one day at a time. Spectrograms of whale sounds for each localisation event were viewed, checked manually and classified based on song structure and song repetition interval. Each localised detection was compared with the previous localisation event and based on the signal characteristics from the spectrogram, as well as the location of the detected vocalisation, it was manually determined whether it was likely to be the same vocalising animal. It was assumed that the average swimming speed of a pygmy blue whale was less than 20 km per hour when comparing locations across longer time scales. A track consistent with a single whale combined with a common ISI amongst songs was used as a criterion for the likelihood of detections being from the same animal. Whale tracks were numbered chronologically and where possible were carried over from the previous sample. Where a consistent signal was lost for more than one sample (greater than 1800 s), or the location of the source did not fit the criterion above, the successive vocalisation was classified as a new vocal animal. The start and end time of each track, length of time calling, song-type, track direction and distance travelled was recorded for each whale track. While each song sequence was unique for an individual whale, many of the sequences were potentially produced by the same whale due to normal breaks in calling. Thus, the manual analysis considers events that occurred independently in time for an individual whale but which may have been replicated at a later time by the same individual.

Quantitative analysis of population wide variability in song structure was carried out using the statistical program R [46]. Repeated measures multivariate techniques were used to test for significant differences in calling duration, song repetition interval and song structure between the sampled years. Unless otherwise stated, errors about mean values are of 95% confidence limits.

## Results

### Song structure and variants

**Fig 5:**
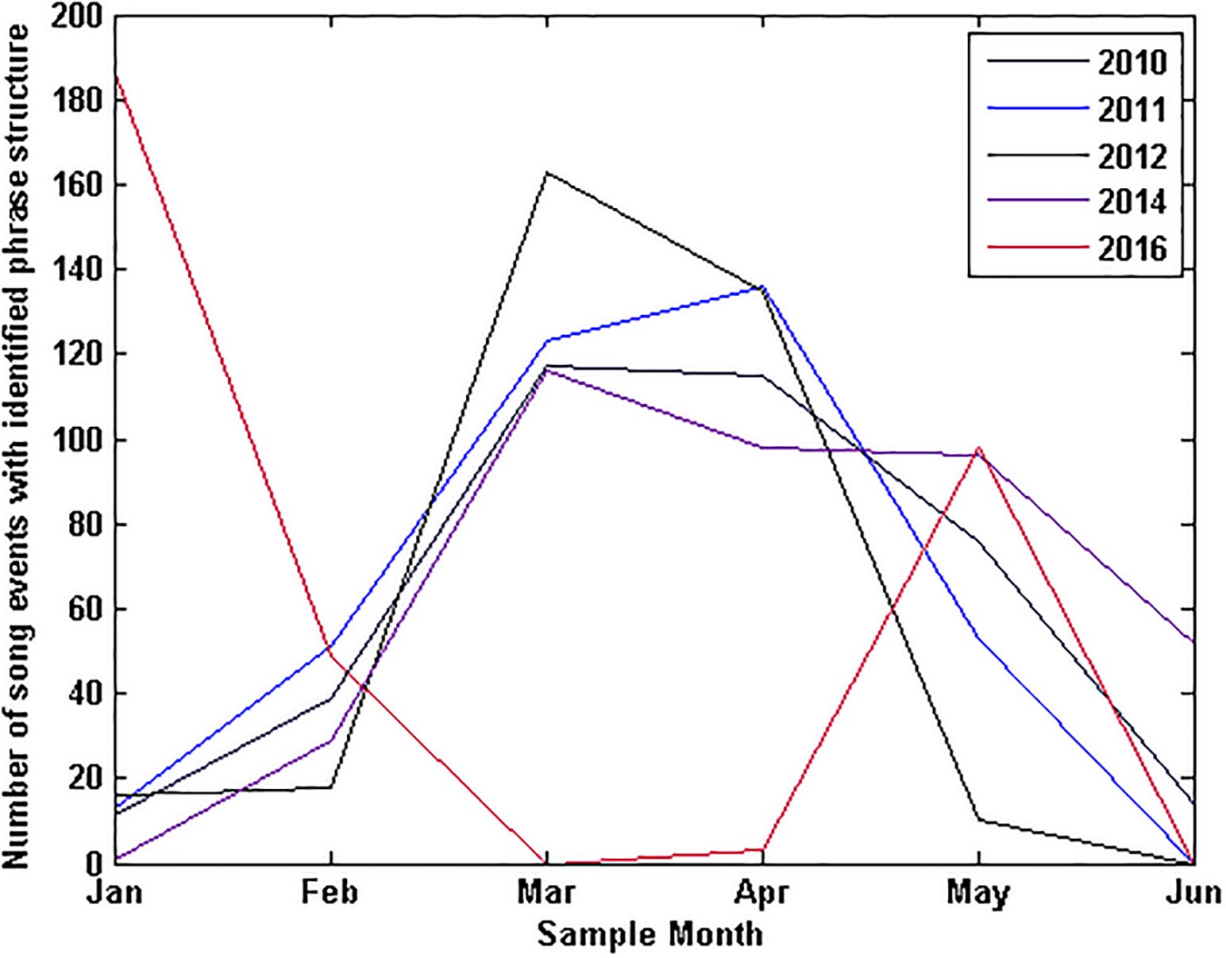
Identified singers per year. Distribution events where song structure was identified throughout the calendar year.

Across the five years of manually analysed samples (2010 to 2016), 2,627 song events from SEIO pygmy blue whales were analysed and identified to song structure. No calls from pygmy blue whales of other Indian Ocean stocks were detected or noted. The highest number of song events analysed were in 2010 and 2011 with 509 and 598 records respectively (Fig 5). Peaks in the number of identified vocal events (Fig 5) were consistent with the northern (February to June) and southern (November to January) migratory pulses of the SEIO pygmy blue whale, which occur along the Western Australian coast at this latitude [15, 16, 47–49]. The largest peak in pygmy blue whale song events was over March and April coinciding with the northern migratory pulse [31] where in some years animals are known to linger in the Perth Canyon engaging in feeding behaviour [37, 31, 50].

The full pygmy blue whale song, or "typical" song, (termed here *P3*) consists of a phrase of three units (sounds) repeated in a sequence with approximately 180–200 s (the value differs amongst years, below) between the start of one phrase and the start of the next (the ISI value, Fig 2a). The first song unit (type I) is the longest with energy centred in the 20 Hz frequency band and harmonics up to 80 Hz. The type I unit starts with a 19 Hz tone that lasts for 21s before jumping to 21 Hz for a further 22 s. This is followed five to ten s later by the type II unit, a frequency modulated upsweep, which for example in 2010 swept upwards from 20 Hz to 26 Hz over a period of 23 s, with energy centred around 24.7 Hz and strong harmonics up to 72 Hz. The last unit of the song, type III, follows ~ 23 s later and is a constant frequency tone between 18 Hz and 19 Hz that lasts between 26 and 28 s. It is accompanied by strong harmonics and a secondary pulsed tone of 60 Hz-65 Hz. Of the total number of analysed song events with identified song-types in the manual analysis, 931 were classified as belonging to the *P3* variant (Table 2). This represented 35.4% of vocalising whales with recognisable song structures. Whilst there were no statistical differences in the proportion of song types between years, there appeared to be a slight decreasing trend in the number of whales producing this song-type through time in the manually processed data.

**Table 2:** Details of pygmy blue whale song variants described from manual analysis.

Code	Description	N	% of identified call type	~ ISI (s)
*P3*	I, II & III repeated ('typical')	931	35.4	180–200
*P3L*	I, II & III with longer ISI to next	11	0.4	220–280
*P3S*	I, II & III with shorter ISI to next	67	2.6	120–160
*P1*	II only repeated	91	3.5	50
*P2*	II & III repeated	1220	46.4	80–100
*P3A*	I, II & III then II & III repeated	263	10	~ 300
*P2A*	*P2* phrase then II repeated	41	1.6	~ 150
*P3B*	*P3* phrase then I & II repeated	3	0.1	~ 280

Given are: the code used throughout; a description of the phrase makeup with the song units involved (types). For *P1*, *P2* and *P3* songs the respective phrases are repeated in a song sequence; the number of occurrences of this song-type; the % of this song-type; and the approximate inter-song interval (s) or the repeat time between type II to the next song-type II unit.

A number of variations to the pygmy blue whale *P3* phrase and song type were found. For the purpose of this study, a phrase was defined as one sequence of units where none of the units are repeated, while a song was two or more repetitions of any phrase structure. Songs that consisted of only one phrase type share the name of the phrase. Common variations included shortening of the phrase to a one (*P1*, unit II only) or two (*P2*, units II and III only) unit phrase and repeating the shortened phrase at a reduced ISI compared to that of the typical song. Combinations of different phrase structures were also identified and termed hybrid song-types. Hybrid song types were named based on the base phrase structure. In 2016 another variation was observed with different units of the song pulsed or broken. Temporal variations to the *P3* song sequence in the form of long or short repetition ISI times were also observed. Details of the phrase and song variants are summarised in Table 2, and discussed below.

The complete *P3* song consists of a repeated phrase with three temporal variants: a) the normal variant; b) a variant with a longer repetition interval than the typical song (*P3L*); and c) a variant with a shorter than normal ISI (*P3S*). The long variation of the *P3* song followed the same basic structure but with 220 to 280 s intervals between the start of one sequence and the start of the next. The extra length of ISI in the song appeared in the length of time between the end of the previous phrase’s type III unit and the beginning of the type I unit of the next phrase sequence. The time between the successive units within the sequence remained consistent between *P3L* and *P3* phrase. The *P3L* variation was identified on 11 (0.4%) occasions making it one of the least common song variants. *P3L* was only recorded in 2010 and 2011.

The *P3S* variation followed the same structure as the *P3* phrase but had only a 120 to 160 s interval between the start of one phrase sequence and the next. The phrase appears to be shortened in the type I unit and the time between the type I and type II units. The time between the end of the third and start of the first unit of the next sequence did not vary greatly from the repeated *P3* phrase structure. The *P3S* song was uncommon and only identified 67 (2.6%) times, occurring in the 2010, 2011, 2012 and 2014 datasets. The production of *P3S* was limited to March, April and May, months with the highest number of song events analysed overall.

The one unit phrase variation (Fig 2b, *P1*) was the simplest variation with the shortest ISI with the first and last units of the *P3* phrase dropped leaving only the type II unit repeated in a sequence with ~ 50 s intervals. The *P1* phrase was one of the least common variations in the manually processed data, identified on 91 (4.3%) occasions. Comparisons across the sample years revealed that the number of *P1* song events appeared to increase between 2009 and 2011. In the manual data, after 2011 the number of whales producing the *P1* song remained relatively constant. Analysis of song structure by month showed the highest number of *P1* song events in March, April and May.

The two unit phrase (Fig 2c, type II and III units repeated, *P2*) was the most common variant of the *P3* phrase in the manual analysis. The subsequent *P2* song sequence is repeated at ~ 80 to 100 s ISI. There were 1,220 *P2* song events extracted from the manually analysed data, which represented 57.7% of sampled song events. The proportion of the *P2* song events remained stable across sample years with peaks in 2010 and 2014 coinciding with peaks in the number of song events recorded.

The appropriateness of song classification was tested with a Fleiss unweighted Kappa analysis on the classification of 22 spectrograms by five untrained observers. The analysis found substantial agreement between observers (*K = 0*.*76*, *z = 25*.*7*, *p = 0*) based on the provided song catalogue. The greatest disagreement between observers was in identifying temporal variations of the *P3* phrase type, *P3S* where the *P3* phrase was repeated with an unusually short ISI value and *P3L* where the ISI between consecutive phrases was unusually long. When temporal variations were ignored, and observers were asked to classify signals into one of the three phrase structures or two song sequence variations, the agreement between observers was almost perfect (*K = 0*.*83*, *z = 23*.*4*, *p = 0*). Based on the results of the Fleiss Kappa Analysis, it can be said that the classifications assigned to phrase and song variations are consistent and appropriate.

Aside from variations in the *P3* song arising from changes in song repetition interval and dropping off different elements, three hybrid song patterns were identified. The *P3A* song pattern (*P3A*, Fig 2d) is a combination of a *P3* phrase followed by a *P2* phrase. The song repetition interval for the *P3* & *P2* song is a combination of the repetition interval for each of the separate sequences, so roughly 300 s as the song comprises a *P3* phrase with ISI of approximately 200 s and a *P2* phrase with ISI of approximately 100 s. The *P3A* song was the most common of the hybrid song patterns with 263 detected whales (10.0%) producing this song variation. The *P3A* variant appeared to be increasing over years, with more than twice the number of occurrences in 2016 (189), compared with the other sample years combined (74). Instances of mixed *P3A* and *P3* song sequences were observed but were rare.

The *P2A* song pattern (Fig 2e, *P2A*) was first found in the 2014 data set and is a hybridisation combining the *P2* (type II & III), followed by the *P1* (type II only) phrase structure. The song repetition interval conforms to that of the *P2* and *P1* phrases with the time between type II units approximately 100 and 50 s respectively, resulting in a total song length of approximately 150 s. The *P2A* song variation is slightly less common than the *P3A* variant with 41 occurrences (1.6%) of this song variant over the sample years. Where followed in time the *P2A* song was consistently repeated (*P2* & *P1* repetitions).

The *P3B* song pattern (Fig 2f, *P3B*) was the rarest song variation detected thus far, only found in the 2010 and 2011 data sets. Along with the *P1* phrase, the *P3B* song structure is the only other variant with the absent type III unit. The song consists of a complete *P3* phrase followed by a two unit sequence consisting of only the type I and type II unit. The length of the song is approximately 280 s, with the *P3* section lasting approximately 180 s and the following two units approximately 100 s. There have been very few occurrences of the *P3B* song variant (< 0.2%).

**Fig 6:**
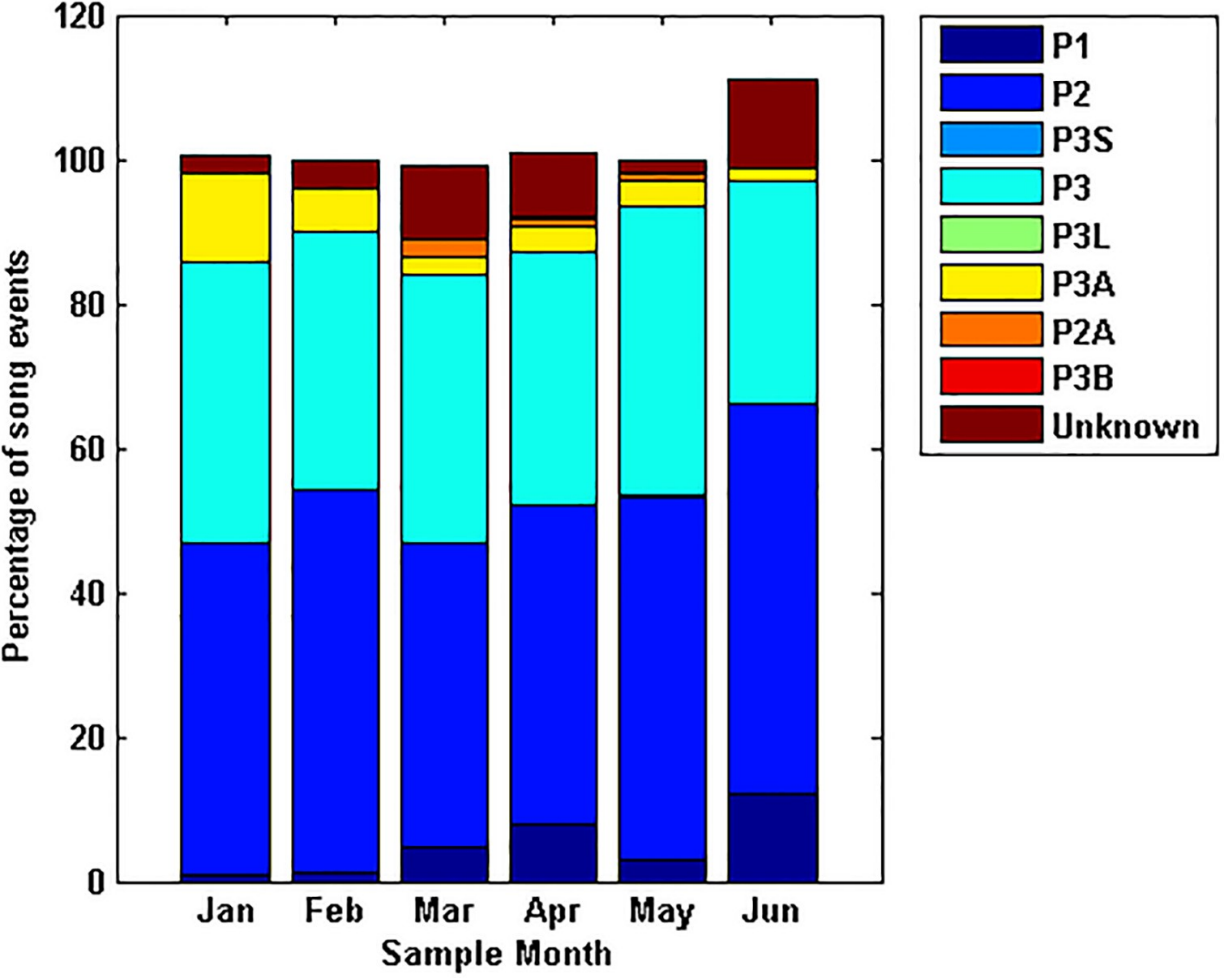
Proportion of song variants each year. Ratio of the number of vocal events displaying each song variant, as a proportion of all identified song events for the sample year. *P3* refers to the three unit phrase song, *P2* to the repeated phrase containing only the second and third units and *P1* to a repeated type II unit only. *P3A* is a song combining the *P3* and *P2* phrases, while *P2A* combines the *P2* and *P1* phrase types. *P3B* is a unique combination of a *P3* phrase and a single unit I and II. Unknown song events are those that are recognisable as SEIO pygmy blue whale vocalisations but the signal is too poor or there are too many overlapping whale calls to identify the song structure.

A breakdown of song variant production across all sample years from the manually processed data shows that the proportion of analysed song events containing the different phrase types remained relatively consistent year to year (Fig 6), though *P3* and hybrid song events appeared to be increasing in the 2016 data set. The greatest diversity in song structure was found in the years with the largest sample sizes (Fig 6). The two and three unit songs were consistently the most prominent song variants in the manual analysis.

**Fig 7:**
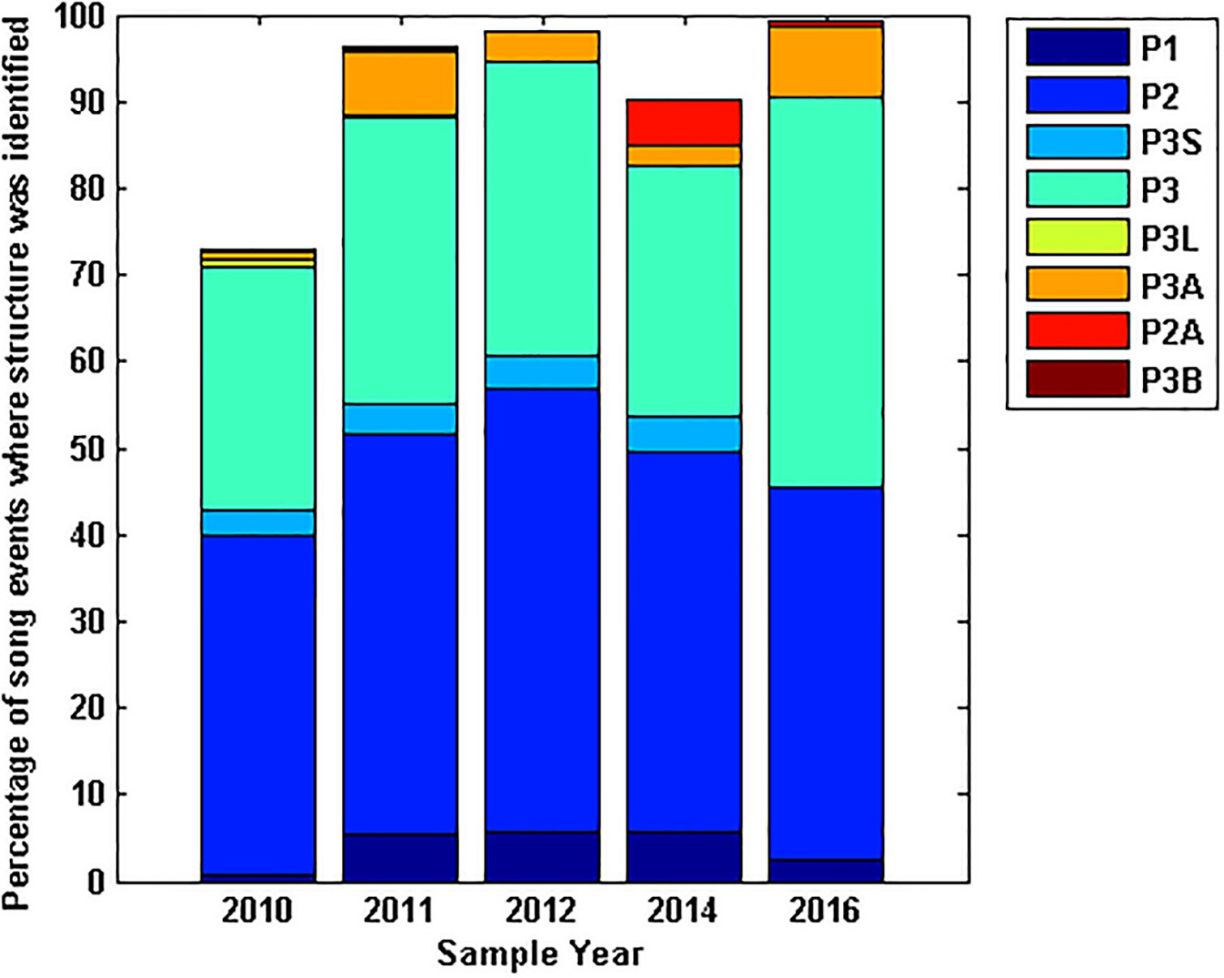
Proportion of song variants within a year. Distribution of song structures throughout the calendar year as a proportion of the total number of song events each month.

There was no trend in the timing of different song variations throughout a season (Fig 7). A larger number of detections of particular song variants in any given month were correlated with a larger sample size. Aside from an exceptionally large number of *P2* songs in 2014, likely due to the high sample number in this year, the number of vocal events identified manually to each song variant in each month of the year was consistent across all sample years.

**Fig 8:**
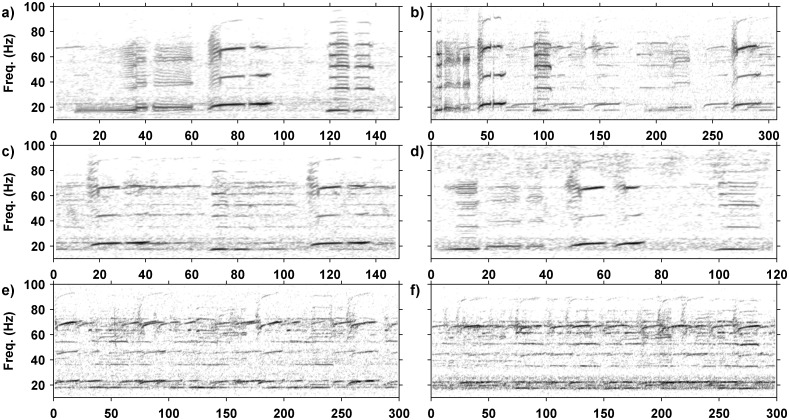
Spectrograms of pygmy blue whale song phrases showing broken units and periods of many singers. Spectrograms of pygmy blue whale song with broken units (a-d) and 5–7 overlapping callers (e-f), made with a 2048 point FFT, 0.8 overlap using a 1 kHz sample rate (0.488 Hz and 0.41 s frequency and time resolution, respectively). The x-axis is time in s, the y-axis is common for all panels. Shown are: a) a *P3* phrase with all three song units broken; b) a *P3* phrase variation with only the type I and type II units broken; c) a *P2* phrase variant with both units broken; d) a *P3* phrase with the type I and II units broken; and e) and f) multiple singers (matched 300 s samples).

**Fig 9:**
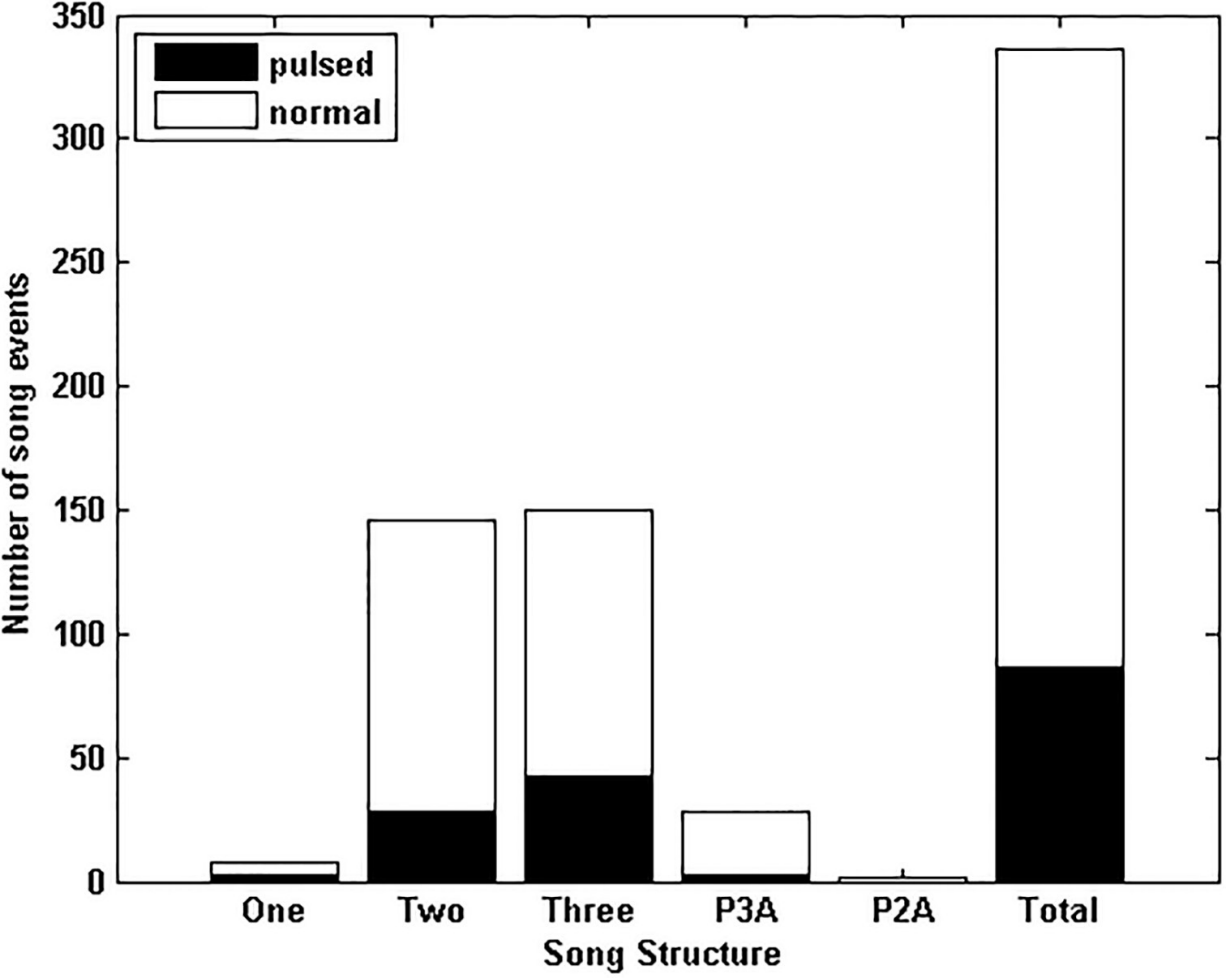
Comparison of phrase-types with normal or broken song units which appeared from 2015 onwards.

Post 2015, variations in pygmy blue whale vocal behaviour have extended to variability in the production of the song units themselves. All three extant song units were observed as being modified with breaks or pauses mid-way through the production of a unit in data from 2016 and 2017. The broken song units occurred across a variety of song structures, with one or more units in the phrase broken in two. The unit containing the break remained consistent within a song event, but varied between song events (ie. Fig 8a–8d). Broken song units occurred in all observed song structures in 2016 and were not observed prior to 2016. The number of song events that were observed with a break in one or more song units compared with similar counts for song events without breaks is shown on Fig 9. Song units containing breaks were present in a large portion (~ 25%) of song occurrences regardless of structure. This trend in the occurrence of broken song units continued into 2017.

It needs to be noted that in the later years it became increasingly difficult to identify individual song events owing to the large number of whales calling and so overlapping song. Examples of samples with multiple callers (between 5–7 callers evident) are shown on Fig 8e and 8f. The high number of singers prevailed for several months and increased across seasons.

#### Feature space song inter-song interval analysis

**Fig 10:**
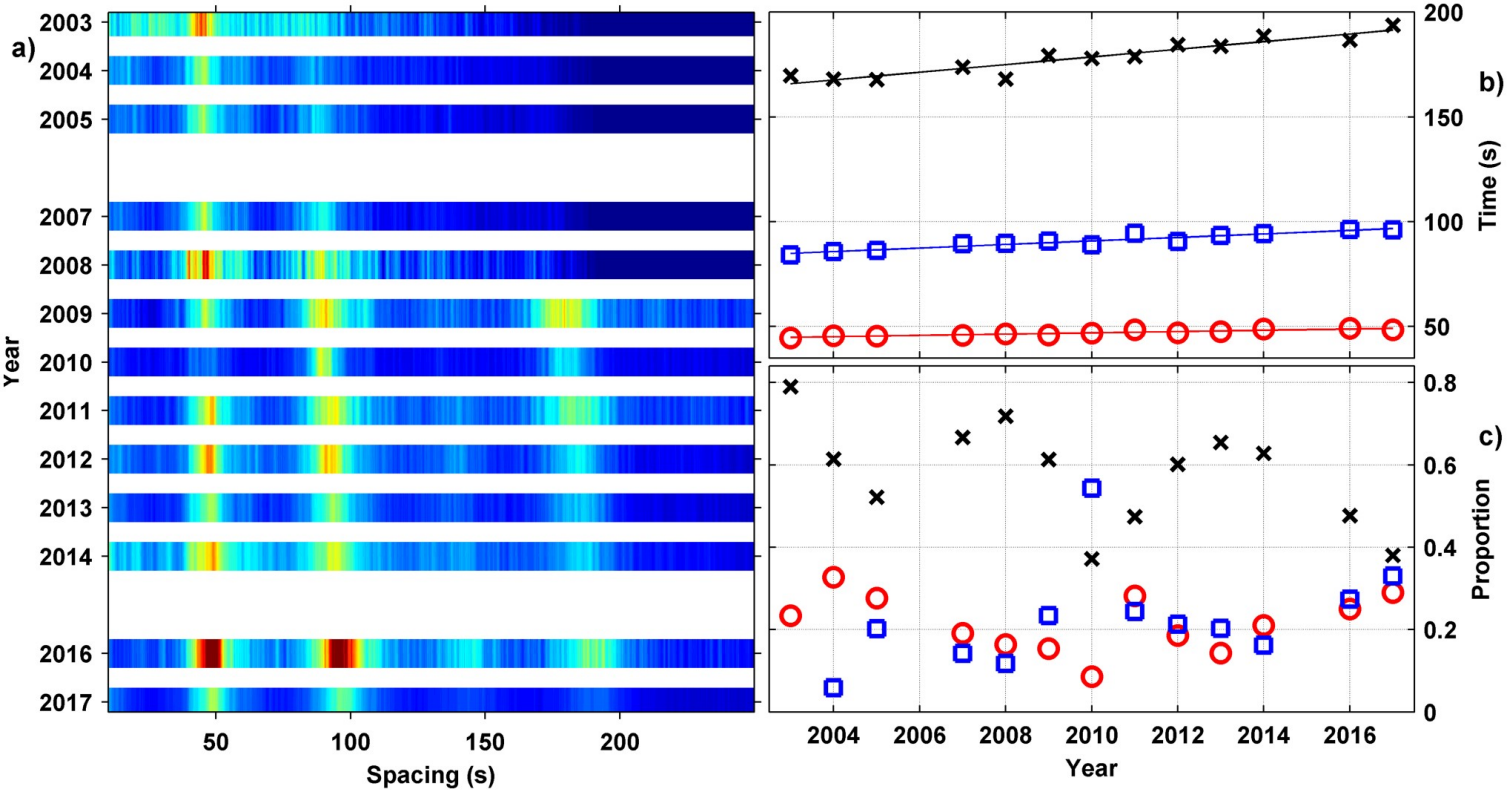
ISI-analysis for all seasons. (a) Density distribution of type II repeat interval for all years (normalised with the colour scale from 0 = blue to 1 = red), (b) peak time interval across years over 30–60 s (red circle, *P1* song ISI), 70–100 s (blue square, *P2* song ISI) and 160–200 s (black cross (*P3* song ISI), and c) proportion of song-repeat values with year (same symbols and colours as b). The solid lines on b) are linear fits. The resolution used in a) was 0.1 s and 0.25 dB.

When a season’s data set was assembled and gridded using the time-level difference analysis, the technique discriminated ISI, as given by the peaks of time between repeat type II song units, for the three predominant pygmy blue whale song-types and multiples of the ISI of each. An example of the 2015 season’s analysis of the ISI, as measured by time differences between consecutive type II units, was shown on Fig 4a, where peaks at the repetition intervals of different song-types appear. By summing values in this time and level difference feature space for a type II unit to the next type II unit, with level differences of < 2.7 dB, the ISI-curves shown on Fig 4b were derived. From the peaks in these curves the time intervals between the *P1*, *P2* and *P3* unit songs were derived. The same analysis is shown for 2003–2017 on Fig 10a, with the ISI values derived from peaks in the summed curves for all years shown (Fig 10b). The thirteen-year analysis shown on Fig 10 involved 119,724 sea noise samples with multiple pygmy blue whale type II units and 545,607 type II to type II time and level difference pairs. The ISI value was increasing over time on Fig 10b for each song-type, at different rates (Table 3). The ISI values for each year along with the ratio of time between the combinations of song variants are listed in Table 4. The ratio between the *P2* and *P1*, ISI (*P2/P1*) was consistent at 1.932 (95%CI 1.9113–1.9527) while the ratio between the *P3* and *P2* ISI (*P3/P2*) was statistically the same, at 1.970 (95%CI 1.9401–1.9999). Thus, the ISI for the three songs (*P1*, *P2 & P3*) were multiples of each other, each ~ 1.951 times longer than the previous song-type according to the statistics. Given that the starting resolution was 0.1 s in the ISI-curve analysis technique, then the ISI of *P2* is approximately twice the length of *P1* while *P3* is approximately twice that of *P2*.

**Table 3:** Fitted curves to ISI-spacing across seasons for *P1*, *P2* and *P3* songs (ISI-curve analysis).

Song-type	Linear Fit	95% CI of coefficient (SE)	Correlation coefficient, *r*^*2*^	F (DF), *p*
*P1* Unit II only	*t* = 0.305 *Y*– 565.5	0.083 (0.038)	0.86	65.9 (1/11), << 0.001
*P2* Units II & III	*t* = 0.843 *Y*—1602.8	0.185 (0.084)	0.90	100.5 (1 /11), << 0.001
*P3* Units I, II & III	*t* = 1.826 *Y*—3491.5	0.435 (0.198)	0.86	85.4 (1/11), << 0.001

Details of linear fits of song repetition interval for the three pygmy blue whale song-types across seasons from the Perth Canyon. The fit values give *t*, the song repetition interval in seconds, for *Y*, the year, with fit statistics given.

**Table 4:** ISI-spacing for the *P1*, *P2* and *P3* songs each year (ISI-curve analysis).

Year	II song (*P1*)	II & III song (*P2*)	I, II & III song (*P3*)	*P2/P1*	*P3/P1*	*P3/P2*
2003	44.55	84.15	169.75	1.889	3.810	2.017
2004	45.65	85.75	172.05	1.878	3.769	2.006
2005	45.55	86.25	167.75	1.894	3.683	1.945
2007	45.75	89.45	173.75	1.955	3.798	1.942
2008	46.55	89.65	168.05	1.926	3.610	1.875
2009	46.05	90.75	179.35	1.971	3.895	1.976
2010	46.85	88.95	177.95	1.899	3.798	2.001
2011	48.45	94.45	178.85	1.949	3.691	1.894
2012	47.05	90.55	184.45	1.925	3.920	2.037
2013	47.55	93.35	183.75	1.963	3.864	1.968
2014	48.85	94.25	188.65	1.929	3.862	2.002
2016	49.25	96.35	186.65	1.956	3.790	1.937
2017	48.45	96.15	193.75	1.985	3.999	2.015
Mean, 95% CI				1.932, 1.911–1.953	3.807, 3.743–3.871	1.970, 1.940–2.000

Song to song repeat interval (s) given by feature space analysis and ratios of these for each year, with: year; (*P1*) time between consecutive type II only songs (s); (*P2*) time between consecutive type II & III only songs (s); (*P3*) time between consecutive type I, II &III songs (s); ratio *P2/P1*; ratio *P3/P1*; ratio *P3/P2*.

To calculate the relative proportions of each song-type from the ISI-curve using equations 1–4 (S1 File) required the mean length of the type II unit, since at least half a type II unit was required for the search algorithm to locate the unit. Based on the time for 90% of the unit energy to pass this length was 20.9 s (95%CI 20.88–20.92) with a median of 21.0 s derived from 499,193 type II signals. The median value was used in the proportion analysis. The mean sea noise sample lengths for each season were listed in Table 1. Using these values, the magnitudes of measured ISI-curves each year and the technique described in S1 File, the relative proportions of each song-type per year were calculated, assuming only songs of the *P1*, *P2* and *P3* types were present (hybrid song-types were ignored) and correcting for additions of multiples of shorter song-types. The trends calculated across time are presented on Fig 10c with statistics listed in Table 5. While the proportion of song-types has varied across the thirteen years, systematically but not linearly, the range is low and the proportions of different song-types fall within a narrow band. Statistics of song proportions (Table 5), have the *P1* (type I) only song present on average across the seasons analysed 21.5% of the time (95%CI 17.30–25.70, all data), the *P2* song (type I & II) present 24.2% (95%CI 16.39–32.01, 2005 on) and the *P3* song (type I, II & II) present 56.0% of the time (95%CI 48.81–63.19, 2004 on). Note the earlier years had relatively short sea noise sample lengths compared to the *P2* and *P3* song length plus fewer whales singing, so early years have been excluded from calculations of statistics of the proportions of *P2* and *P3* songs.

**Table 5:** Proportions of *P1*, *P2* and *P3* song-types given by ISI-curve analysis.

Song	Min.–max.	Mean, 95% CI, (median)	Years included (N)
*P1* Type I only	8.6–32.7	21.5, 17.30–25.70 (21.0)	2003–2017 (13)
*P2* Type I & II	11.8–54.3	24.2, 16.39–32.01 (21.3)	2005–2017 (11)
*P3* Type I, II & III	37.2–71.8	56.0, 48.81–63.19 (60.7)	2004–2017 (12)

Statistics on proportions of each song-type as given by the feature space analysis of type II to type II time-level differences.

## Discussion

The song structure of SEIO pygmy blue whale has been shown to be variable and changing across time, while still retaining its uniqueness when compared with the song types of other Southern Hemisphere pygmy blue whale stocks. The fundamental song structure is three units repeated in a phrase (*P3*), but with a further two common variations (*P1* and *P2*) in which not all units are repeated per phrase, sections where phrases combining different combinations of units are repeated and recently, the alteration of units by splitting them into two sections.

The high level of agreement in the classification of different song events by multiple untrained observers provides support for the classification of phrase and song structures outlined in this analysis. The temporal variations of the *P3* song type, delineated from *P3* by a longer or shorter than average phrase repeat interval, proved to be the most difficult for untrained observers to identify. Removal of the temporal element of song event classification resulted in a near perfect agreement between observers. This in part may be due to an inconsistency in the length of sample times which often precludes the recordings of multiple phrases for longer song variants. As such it is recommended that longer recording times be utilised in future studies to capture the variability in phrase repeat times. Additionally, further investigation into the temporal variability of song structures may help to better identify temporal song and phrase variants.

The observed changes in song structure were in the form of variations to: 1) the structure of units with broken or split units observed in recent years; 2) variability in phrase composition where different units were omitted (*P1*, *P2 or P3*) or where a consecutive song had a different arrangement of units to the previous phrase-type (hybrids); 3) the duration of a phrase cycle; and 4) the interval between consecutive phrases within a song, which increased yearly. In the context of this discussion, the *P1*, *P2* and *P3* phrase type refer to singular sequences of these phrases while the *P1*, *P2* and *P3* song variations refer to repeated sequences of the respective phrase types. Hybrid song types are those which alternate between two different phrase structures. There are a number of adaptive processes by which song changes can occur and a number of factors that may be driving these changes. Gradual changes to song structure over a long period of time may be attributed to slow evolutionary processes such as genetic or cultural drift [16], whereas rapid changes to characteristic vocalisations may reflect changes in environmental or physical conditions [51]. Further short-term shifts in the structure or content of songs may be indicative of the social or behavioural context of the caller [28]. It must be understood in interpreting song variation that at this point in time, we have no information as to the sex of singers for pygmy blue whales and it would be wrong to automatically assume all singers are males. However, given the large body of information on song production in other species of whale, along with other taxa, it is suspected song may have some function in mate attraction and selection [4, 52, 53].

It is tempting to relate the observed long term, linear decline of the frequency of the type II unit in pygmy blue whale songs (a decrease of 0.12 Hz/year in the call fundamental frequency [47]), with the long term, linear increase in the song repetition interval as found here. There is growing evidence to support a relationship between annual changes in frequency and ISI, particularly in the songs of fin whales [52, 53]. However, we could find no simple association between increased ISI values and a decrease in call frequency across time, suggesting further investigation is needed to identify any relationship between the frequency and temporal domains of song production for the pygmy blue whale. Song structure is likely to confound any attempt to relate the two parameters in this study as tonal frequency is measured from the type II song unit and not the overall song, while ISI takes into consideration the repetition interval for the entire phrase sequence. The findings of this study indicate that the rate of change in ISI is variable between song variants with an average increase of 0.30 ± 0.083 s/year, 0.84 ± 0.185 s/year and 1.73 ± 0.466 s/year for the *P1*, *P2* and *P3* songs respectively. How or if the increase in ISI through time observed here and the decrease in call frequency through time observed by [47] relate to each other is not yet clear.

Changes to song repetition interval may result from an increase in ambient noise, primarily here from the chorus of other pygmy blue whales singing in the same area. In the most recent data sets the largest obstacle to identifying the song structure of vocalising animals in the manual analysis was other vocalising animals which flooded samples with pygmy blue whale signals. As population numbers increase ([48, 49] for EIO pygmy blue), it may be a natural adaptation for song repetition intervals to increase. Whether this may be because animals do not have to call as frequently to attract a mate, or the benefit of producing more signals in a shorter time period is outweighed by the energetic cost, or competition due to increases in vocal activity of other animals, would require more investigation. Such density dependent singing dynamics have been studied widely in other taxa such as birds and frogs [6, 7, 54, 55]. There is also the possibility that changes in the body size of individual whales may have occurred post whaling, with the proportion of larger animals increasing as the population increases. An increase of larger animals may correlate with the observed increase of ISI time separation, but, as we have no data on comparative body size across years this cannot be verified.

The three primary phrase-types defined all contained the type II unit, indicating that this unit seems to underlie song structure of the SEIO pygmy blue whale population. Intriguingly, the time between consecutive songs seemed to almost double for renditions of each song-type (Table 4). Given that the resolution used in the analysis to obtain these ISI values was 0.1 s, the ratios of ISI between the three song-types is close to two for *P1* to *P2* and *P2* to *P3*, ISI values respectively. This suggests that inter-song interval may be set by a common oscillator or internal clock, sampled at integer multiples. Further to this, vocal animals displaying the hybrid song structures demonstrated a strict timing conformity with the second phrase of the sequence lasting half the time of the first phrase. For instance, an animal producing a *P3A* song with the first *P3* phrase lasting ~ 180 s was followed by a *P2* phrase of ~ 90 s length, again suggesting a fixed ratio relating to phrase intervals.

The proportion of each song-type calculated from the manual analysis of song events and the ISI-curve analysis technique utilising time and level differences between all type II units in a sea noise sample, differed significantly (4.3, 52 and 44% for *P1*, *P2* and *P3* song occurrence from manual measures, and 21.5, 24.2 and 56.0% for *P1*, *P2* and *P3* song occurrence respectively from the ISI-curve analysis when averaged across all seasons). The ISI-curve analysis method was largely independent of biases, although: 1) the curves produced for each song-type will be slightly smeared due to different ISI values between song events; and 2) was sensitive to sample length pre 2009 (samples < 300 s). For the samples of shorter length the proportions of the longer song-types which could be detected in that sample length dropped significantly, thus lowering curve peak values and so increasing errors in determining curve peak values and baseline noise levels (since the peak level may have been close to that of background noise or the background noise was sloped downwards for longer ISI times as it approached the sample length). But, the trends in the ISI-curve analysis technique were largely consistent across seasons which were treated independently, the expected proportion of third multiples of the *P1* ISI were identified correctly by the technique, and the equations used gave the correct ratio of song proportions within < 1% when comparing derived proportions from set proportions using a simulator (S1 File). The manual method will be subject to biases, primarily because the songs that were analysed had few background callers present to reduce confusion in following the same vocalising animal. There may also be inherent biases where during periods with multiple singers present, animals are more likely to produce songs similar to the other vocalising animals resulting in a shift towards one particular song structure. The proportions derived from the ISI-curve analysis would be preferred when using the values to manipulate song counts into abundance measures.

Either analysis technique indicates that song occurrence is not a direct reflection of calling whale abundance. The presence of shorter ISI values than the *P3* phrase-type means that simply counting the presence of type II units across time (song/time) will not directly equate to the number of animals present at the time. The ISI-curve analysis shows that while across the 13 years of sampling available the relative proportions of each song-type does not change in any systematic way, suggesting phrase production per unit time may be comparable for relative abundance, the proportions have considerable variation in ranges amongst years of up to ± 8% for the 95% CI of any song-type (Table 5). Thus, comparing rates of phrase per unit time as given by the presence of one call unit, may not be directly valid without correcting for changes in ISI values and the relative proportion of song-types. For using song as a measure of abundance in pygmy blue whales then some discrimination of song-type (*P1*, *P2* or *P3*) needs to be made. One technique utilised by the authors is to split a sea noise sample into time windows less than the *P2*, ISI (96.4 s in 2017, 70 s is a window length commonly used), count the number of type II units within each window and use the maximum value in any window as an estimate of the number of vocalising whales. Using the manual ISI analysis song-type proportions suggest this is correct for 96% of songs, while using the ISI-curve analysis technique for proportions of song-types averaged across seasons suggests this is correct for 80% of songs.

The large degree of variability in ISI values demonstrates that differences exist in timing and phrase production even within similar song structures. This indicates the possibility that variability in phrase structure may be attributed to individual callers and reflects important social information such as identity, sex or size [29, 56]. The importance of song in individual identification has been explored in bird song as well as the signature whistles of dolphins and dialects of killer whales [57–58]. However, studies of individual variability are more limited in baleen whales. The ability to identify individual animals by their song would likely be beneficial in any population of social animals where the mechanisms and cognition exists to allow this to happen. It is unclear whether individualisation in bird song results from physical or social differences in song learning and production, though studies in cetaceans suggest that individualisation may be a social process [58]. The consistency in the proportion of detected vocal animals producing the less common song varieties would suggest that there may be an individual or familial link between the song variant produced and the vocalising animal. Familial linkages can occur through genetic or learned processes, which are often intrinsically entwined in maternal animals that exhibit social behaviour [59–61].

Patterns in the timing of song production and as seen in the ISI analysis are reminiscent of the phonology of speech and song in other species. Phonology, or the arrangement of sounds within a language, allows for the interpretation of different song elements based on a hierarchical context [41, 62]. The ability of animals to display elements of complex phonology has been demonstrated previously in studies of song-bird populations [41, 62, 63]. The findings suggest that mechanisms underlying complex phonology likely evolved separately and prior to the human linguistic traits of semantics and syntax [62]. It is therefore likely that these traits will be present in the communicative abilities of other evolutionary lineages such as cetaceans.

For a number of cetaceans, it is nearly impossible to separate genetic linkages on the maternal side and cultural processes as the cause of particular behaviours that are passed from mother to calf [8, 12, 14, 32]. In baleen whales, song is believed to be a male only phenomena though it is unclear whether it is learnt or inherited and it must be reiterated that the sex of pygmy blue whale singers has not been confirmed. The existence of a large number of song variants combined with the low genetic diversity of pygmy blue whales [64, 65, 66], suggests that it is unlikely that song variants are linked to genetic drivers alone. Genetic processes are slow to act as they occur over the life cycle of an animal. Given the rapid appearance of phrase and unit variations, which seem to appear within a season, it is implausible that genetic processes are responsible for the variability in song production.

Culture is recognised as a driver of behaviour in cetacean species. Culture relies on the social and familial networks of a species and in turn is a driver of social behaviours of a species such as the production of song [11, 15, 19, 59, 67–69]. The hybrid pygmy blue song patterns, as well as the broken song units that have appeared post 2015, reflect an increase in complexity of song structure setting them apart from the *P1* and *P2* variants of the typical, *P3* phrase song. Increased complexity within songs is thought to be a reflective of cognitive fitness, which may be a favourable trait for sexual selection [6, 55, 70, 71]. Innovation is a cultural process whereby an individual makes a change to the song structure and this change is then copied by other whales and can spread through the population [11, 15, 20, 30, 41]. Vocal learning is the primary mechanism by which changes to song are proliferated throughout the population as well as the means by which juveniles learn the characteristic vocalisations of the population [19, 23, 26, 58]. Similarly, errors in vocal learning can result in variations to song structure, which may then be passed on to others within the population [26, 30, 34, 58, 68]. Cultural processes are generally widespread as is seen in humpback populations where changes proliferate through the population [68]. There may be selective pressure for song variation and diversity within the population with females displaying a preference for novel or complex song-types, as is frequently the case with bird song [6, 55, 62, 72, 73]. Innovation and cultural proliferation would be a more reasonable explanation for the rapid inclusion of unit variations in a significant portion of the pygmy blue whale song phrases post 2015. The fact that broken units represent such a high level of variability suggests a more complex mechanism of song learning and proliferation within the population.

Whilst not energetically costly, singing represents a cost to the animals in terms of the time involved, as it is assumed to preclude other behaviours such as feeding [74, 75]. As such the time demands of singing must be balanced with any benefits it provides such as increased reproductive output [74, 75]. Where male whales dominate singing, song is presumed to have a role in attracting female conspecifics as well as in competing with other males in the area [70, 75]. Sound source level analysis has revealed that the first unit of the *P3*, SEIO pygmy blue whale phrase-type is the least intense and thus in high levels of ambient noise is the hardest to detect [5, 37, 40, 47, 76]. The second unit is the most intense making it the easiest to detect [40, 76, 77]. The Perth Canyon is becoming noisier, largely as a result of the increased number of pygmy blue whale vocalisations [36, 78]. Thus, focusing time and energy on producing the song units that are best transmitted among high levels of ‘noise’ and removing the lower level signals could potentially provide a benefit with the individual more likely to be heard by females in the area, as well as by keeping the ‘noise’ down. Studies of humpback whale calling behaviour suggest that female whales prefer more complex songs [4, 17, 70, 79] which is what appears to be happening with the hybrid song-types and unit variations. There have been observations for other mammals of the ability to change song structure dependent on environmental conditions [2, 53]. For instance, audience effects (increasing source levels) have been observed in the communication of close range gorilla calls whereby vocalisations were changed dependent on the distance of the caller to the receiving animal [80]. Such a capacity to adapt vocalisations based on target audience and environmental conditions would likely prove beneficial to cetacean species as well, especially as their acoustic environment becomes more complex [36, 78]. From the data analysed here, it would not appear that there are distinct patterns in the production of particular phrase and song varieties at different times of the year or even within single days as multiple variants were present within one 24 hour period. Whilst this does not negate the potential existence of a relationship between environmental conditions and song production, a more detailed analysis of physical ocean properties, ambient noise and dominant song varieties would need to be conducted to look for any relationship.

One of the most significant findings of this study is that variability exists in the characteristic song of the SEIO pygmy blue whale subpopulation. Song structure has previously been used as a diagnostic tool to separate populations of pygmy blue whale (McDonald). Variability in what were previously considered to be static signals raises questions as to the validity of song structure as a diagnostic for sub populations. Consequently, it is recommended that a global study on variability within and between the vocalisations of all sub populations of pygmy blue whale be conducted.

## Conclusion

Through long term passive acoustic monitoring we found three distinct variations to the South Eastern Indian Ocean pygmy blue whale phrase structure in the Perth Canyon, Western Australia, and a further three song pattern variations. Within these phrase structures there exist variations in the inter-song interval resulting in two further temporal variations on the three unit phrase structure. Further, the most recent data sets include variations to the units where they are split, or ‘broken’, within the existing song structures, which adds an additional level of complexity. The mechanisms behind the increase in song diversity are unclear though the rapid appearance of new phrase variants that represent progressive changes to the original phrase structure is consistent with cultural evolution. Such rapid change, with new variants appearing within a migratory season, indicate that the levels of variability cannot be attributed to genetic processes. Variability in song and phrase structure is not prolific throughout the population with all the variations present within one year. This sets pygmy blue whales apart from the well-studied humpback whales where changes in song structure are generally propagated through the entire population and supersede earlier song-types. This raises the question as to whether physical environmental conditions may influence song production as has been documented for other baleen whale populations [53]. Peaks in the number of calling animals displaying various phrase-types and the relative stability of the number of detected song events with rare structures across the sample years suggests that song variation may be linked to individual animals though further studies are needed to explore this. It is unclear whether physical environmental processes (noise produced by the whales singing) or cultural processes are at play as the concept of culture has only been explored in odontocete and humpback whale populations. There is also the potential for a level of signal plasticity to exist allowing for context-specific production of vocal cues. Further studies utilising passive acoustic techniques and visual observations, as well as genetic analysis are recommended to elucidate the function of pygmy blue whale song and the driving forces behind changes in phrase structure that are directly translatable to song structure. It is also recommended that a detailed study of fine scale vocal parameters, including temporal variability be conducted to identify the level of variability between vocalising animals. Finally, it is evident that there is a need for comparative studies between pygmy blue whale populations to assess widespread variability in song production.

## Supporting information

S1 File Example ISI-curve’s for seasons 2007 (blue curve for raw data, red curve for smoothed curve) and 2012 (magenta for raw data, black for smoothed curve) with details of ISI feature space analysis techniques. (DOCX)

S1 Data Manual analysis data. (XLSX)

S2 Data ISI-curve data. (TXT)
