# Supplementary material for: Song variation of the South Eastern Indian Ocean pygmy blue whale population in the Perth Canyon, Western Australia
Source: PLoS One. 2019 Jan 22;14(1):e0208619. doi: 10.1371/journal.pone.0208619 (PMC6342329; doi:10.1371/journal.pone.0208619)
Supplement: S1 File — (DOCX) [file pone.0208619.s001.docx]

Supporting Information S1

# Song variation of the Eastern Indian Ocean pygmy blue whale population in the Perth Canyon, Western Australia

Use of inter-song interval curves to define the proportion of pygmy blue whale song- types.

Capri D. Jolliffe^1^, Robert D. McCauley^1^, Alexander N. Gavrilov^1^, K. Curt S. Jenner^2^, Micheline-Nicole M. Jenner^2^, Alec J. Duncan^1^

^1^ Centre Marine Science and Technology, Curtin University, Perth, Western Australia

^2^ Centre for Whale Research (WA) Inc., Fremantle, Western Australia

This analysis resulted from calculating the time spacing and level difference between all combinations of type II song units found within a sample and appending these pairs for all samples within a season. These time and level difference pairs were gridded, then summed for all level differences < 2.7 dB to give an “ISI-curve” which displayed peaks at the inter-song-spacing (ISI) for the three major song varieties (*P1, P2* and *P3*). All song types had the type II unit and there was only one type II unit within each song type. Obtaining the ISI value for each curve was relatively simple and was simply the time values at each of the curve peak’s. Obtaining the proportion of a song type was more complex and is described here. The magnitude of peaks in the ISI-curve was dependant on three factors: 1) the noise levels resulting from random spacing of type II to type II units (different individuals singing) or false detections; 2) the prevalence of a song type producing the peak (plus multiples of shorter song types adding to this where applicable); and 3) the expected occurrence of a song length in a sea noise sample length. Examples of ISI curves for an early season of 2007 with shorter sea noise samples (205 s) and a later season (2012) with longer sea noise samples (307 s), is shown on S1 Fig. For a short sea noise sample length, the curves tail off at the longer ISI values as the expected number of long song types which fit into the available sample length drops rapidly.

It transpired that the shorter song type ISI values produced multiple peaks in the ISI-curve and were an integer multiple of the longer song types, thus adding into the peaks for the longer song-type, ISI-curves. In the methods here we initially define the song proportion analysis by assuming the peaks in the ISI curves are independent or occur at times which are not multiples of each other. The overlapping ISI values, or shorter song types which do have ISI values which are integer multiples of longer song types, are then dealt with.


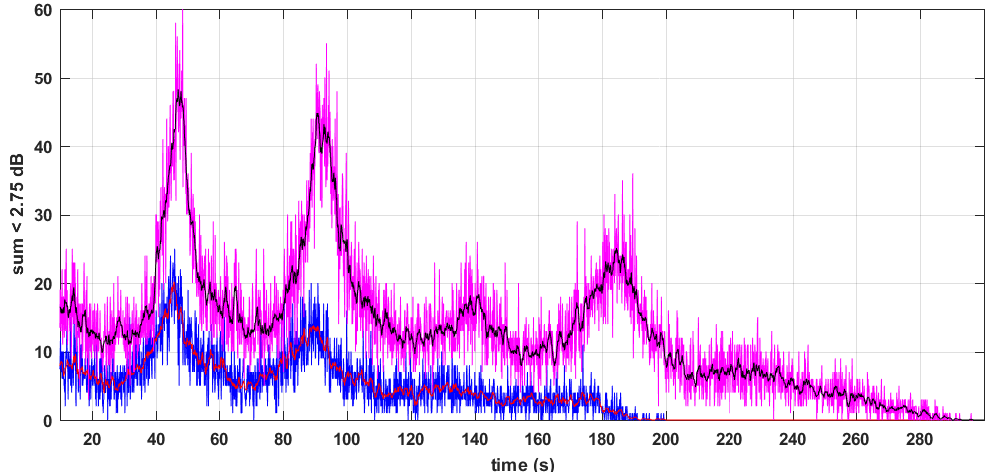


S1 Fig. Example ISI-curve’s for seasons 2007 (blue curve for raw data, red curve for smoothed curve) and 2012 (magenta for raw data, black for smoothed curve).

The first step in analysis was to account for “noise” of a peak in the ISI-curve. Noise levels were calculated by obtaining the base noise level at the centre of the respective ISI peak by interpolating between the low values either side of the peak. In the example shown on Fig 4 the noise levels were flat, but for the shorter sea noise samples (pre-2009, Table 1 or 2007 data, S1 Fig) the base noise levels were sloped downwards for longer song types reflecting the decreasing number of songs of longer length expected in samples of shorter duration. Once noise level values for each respective peak were derived they were subtracted from the maximum value of each peak in the smoothed ISI-curve (smoothed using a running linear fit, 5 points either side of point in question). The integer number of songs (*N_m_*) which can be detected in the mean sample length (*T* in s, Table 1) was then calculated. The search algorithm used to locate type-II units required at least half of the length of the type II unit (*L* in s), thus the mean sample length for each deployment was reduced by *L*/2 to account for this. The maximum number of full song-cycles (*N_m_*) which will fit into a sample is:

(1)

where *S* is the song repetition interval (s) derived from the type-II to type II, time-level difference analysis described above for the respective song type and *floor* rounds to the lowest integer value. The value *N_m_* is not the expected number of song cycles, which may be detected within a sample of given length, which is defined by the two equations:

(2)

which gives *c*, the available sample length minus the time for *N_m_* - 1 full song cycles. The expected number of song cycles (*α*) which can be detected in the sample of length *T* for a song-cycle of length *S*, is then:

(3)

For the simplest situation where we assume no overlap of multiples of one song type with the ISI of another song type, the value of *α* can be used with the peak y-values derived from ISI-curves (corrected for noise) for all song-types, to give the relative proportions (0-1, *r_i_* where *i* denotes the song type) of each of the three main song types within a season as:

(4)

where *Y_i_* is the y-value, corrected for background noise addition, found at the peak of the ISI-curve from the time-level difference analysis, and *i* refers to the song type. As mentioned above, it transpired that multiples of the ISI values for the shorter song types added to the *Y* values for the *P2* and *P3* song ISI values. The ISI of the *P1* song was just under half of the ISI of the *P2* song and one quarter of the *P3* song. This meant that the ISI value for *P1* to *P1* to *P1* songs (*P1* x 2) calculated by the ISI-curve technique added into the *P2* to *P2* (*P2* x 1), ISI-curve. To account for this, the value of *Y_i_* used in Equation 4 for the second song type (*P2*) had to be reduced (to *Y_2_*) by the expected proportion of *P1* x 2 songs. The value to reduce it by, was calculated from the expected number (α_4_) of a song with ISI value of length *P1* x 2 determined using equations 1 & 2, divided by the expected number of the *P1 to P1*, ISI (α_1_) and to multiply this by the *Y_1_* value (corrected for noise) used in equation 4, for the *P1 to P1* song, ie. , so (Equations 5 & 6). This corrected value of *Y_2_* was then used in equation 4.

Similarly, the *P1* to *P1* to *P1* to *P1* to *P1,* (*P1* x 4) multiple added into the *P3* curve peak, as did the *P2* to *P2* to *P2 (P2 x 2)* multiple, so these also had to be removed in the same fashion, from the *P3* peak value. The magnitude of the *P2* x 2 value which contributed to the *P3* curve magnitude used the *P2* x 2 curve peak magnitude after correction for the *P1* x 2 contribution.

As an initial check on the use of the y-values, or magnitudes, in the ISI-curves for calculating relative proportions of song we can compare a multiple of the *P1* song (*P1* x 3) with the *P1 to P1*, ISI. It can be seen that a peak occurred at ~ 145 s on the ISI curve of Fig 4b or S1 Fig above, which did not overlap any of the other song types or multiples of these. This was the *P1* x 3 peak. If the logic of equations 1-3 were correct then the y-value (noise corrected) of the *P1 x 1* and *P1 x 3* peaks divided by the expected number of each call type should be the same, or if these are divided by each other they should give one, which they did within the error bounds, at 0.95 ± 0.17 (± 95%CI) when dividing the *P1 x 1* by the *P1 x 3* result.

As a second check on the validity of Equations 1-6 and to provide error estimates on the proportions of each song type calculated per season using the ISI curve technique, simulated data sets were built and the analysis using equations 1-6 as used on the real data sets applied. The proportions of songs set in the simulated data set were compared with the song proportions derived using the analysis technique. The simulated data used 100 model runs with overlapping ISI values (ie. the longer song ISI values were multiples of the shorter song-type ISI values). Each run: used 1000 samples each 500 s length; had 3000 songs in total set in ratios of 10.8-29.6% of *P1* songs, 10.2-29.7% of *P2* or 44.8-77% of *P3*, such that the proportions (*P1 + P2 + P3*) added to one for each run, the ratios were randomised within this range for different runs; between 1-3 callers per sample (where callers were present, many samples in a model run had no callers) were allowed such that the correct proportions of song types were maintained; used call ISI values of 45 (repeated *P1* song), 90 (repeated *P2* song) and 180 s (repeated *P3* song) or 50, 100 and 200 s (repeated *P1*, *P2* and *P3* song, respectively), with the ISI value of each 'individual' varying by ± 0.5 s in a random fashion; setting the start-point of a song sequence as a random proportion of the ISI increment for that individual from the start of the sample; and used a level range of from 1-15 dB with consecutive songs of the same individual allowed to randomly vary in level by ± 1 dB. This analysis gave error estimates (root-squared errors of hard fixed song proportions compared with derived proportions) for all song types combined at 0.8% (± 0.07% 95%CI or SD of 0.67%, N=300), or for the *P1* song proportion at 0.5% (± 0.11% 95%CI or SD = 0.54%, N=100), *P2* song proportion at 0.9% (± 0.14% 95%CI or SD = 0.71%, N=100) and the *P3* song proportion at 1.0% (± 0.14% 95%CI or SD = 0.69%, N=100). The comparatively low error estimates between song proportions set and derived from this analysis plus the correct prediction of a multiple of the *P1* song type (*P1* x 3) in real data sets show the ISI-curve analysis technique to be valid in predicting song proportions.
